# Dysmagnesaemia and Critical Illness Outcomes

**DOI:** 10.1186/2197-425X-3-S1-A499

**Published:** 2015-10-01

**Authors:** LE Homer, C Edmark, KM Mogensen, FK Gibbons, KB Christopher

**Affiliations:** University of Liverpool, Liverpool, United Kingdom; Karolinska University Hospital, Anaesthesiology, Critical Care and Surgical Services, Solna, Sweden; Brigham and Women's Hospital, Department of Nutrition, Boston, MA, USA; Massachusetts General Hospital, Pulmonary and Critical Care Medicine, Boston, MA, USA; Brigham and Women's Hospital, Renal Division, Boston, MA, USA

## Introduction

The true impact of Magnesium derangement is unknown in critical illness. Conflicting reports in small studies have been published regarding mortality and dysmagnesaemia in critical care.

## Objectives

We hypothesized that hypomagnesaemia would be associated with adverse outcomes following critical care.

## Methods

We performed a two center observational study of patients treated in medical and surgical intensive care units in Boston, Massachusetts. All data was obtained from the Research Patient Data Registry at Partners HealthCare. We studied 81,061 patients, age ≥ 18 years, who received critical care between 1997 and 2012 and who survived 72 hours following ICU admission. We excluded patients with End Stage Renal Disease. the exposure of interest was the lowest serum Magnesium measured in the 24 hours prior and 72 hours after ICU admission categorized a priori as < 1.4 mEq/L, 1.4-1.7 mEq/L and >1.7 mEq/L. the primary outcome was 30-day all-cause mortality determined by the US Social Security Administration Death Master File. Adjusted odds ratios for 30-day mortality were estimated by multivariable logistic regression models with inclusion of terms for gender and the Acute Organ Failure score [[1]], a validated ICU risk-prediction score inclusive of terms for age, race, comorbidity, patient type (surgical vs medical), sepsis and acute organ failure.

## Results

The cohort (N = 81,061) had 58% men, 80% white race, 52% surgical patients, 12% with sepsis, and a mean age of 62 years. 30-day mortality was 9% and 30-day hospital readmission was 14.2%. in patients with magnesium < 1.4 mEq/L, 1.4-1.7 mEq/L and >1.7 mEq/L the 30 day mortality rate was 9%, 8% and 10% respectively. the adjusted odds of 30-day mortality in patients with Mg < 1.4 mEq/L was 0.94 (95%CI 0.87-1.01) and with Mg >1.7 mEq/L was 1.22 (95%CI 1.15-1.29), both relative to patients with Mg 1.4-1.7 mEq/L. the AUC for the model was 0.79 indicating good discrimination for 30-day mortality. Further, in medical ICU patients (N= 39,041), adjusted odds of 30-day mortality in patients with Mg < 1.4 mEq/L was 1.02 (95%CI 0.92-1.13; P = 0.70) and with Mg >1.7 mEq/L was 1.45 (95%CI 1.35-1.56; P < 0.001), both relative to patients with Mg 1.4-1.7 mEq/L. Additional adjustment by acute kidney injury (RIFLE class injury or failure) or chronic kidney disease stage did not alter the Mg-mortality association. Finally, in patients with nutrition status determined (N = 6,923) there was no significant difference in serum Mg relative to nutrition status (ANOVA P-value < 0.001).

## Conclusions

In this large population of critically ill adults, hypermagnesaemia near ICU admission in medical ICU patients is associated with short term mortality. Hypomagnesaemia does not appear to be associated with adverse outcome.

## References

[1] Elias KM, Moromizato T, Gibbons FK, Christopher KB (2015). Derivation and validation of the acute organ failure score to predict outcome in critically ill patients: a cohort study. Crit Care Med.

